# Supplementary material for: Socioeconomic inequalities in abdominal obesity among Peruvian adults
Source: PLoS One. 2021 Jul 21;16(7):e0254365. doi: 10.1371/journal.pone.0254365 (PMC8294571; doi:10.1371/journal.pone.0254365)
Supplement: S1 Table — (DOCX) [file pone.0254365.s001.docx]

| **S1 Table. Prevalence of abdominal obesity in men (n = 26,789) and women (n = 35,349) according to socioeconomic characteristics (ENDES 2018-**  **2019) and based on the guidelines of the Third Adult Treatment Panel (ATP III) and the Latin American Consortium of Studies in Obesity (LASO).** | | | | | | | | |
| --- | --- | --- | --- | --- | --- | --- | --- | --- |
|  | Abdominal obesity prevalence by ATP-III (%) ○ | | |  | Abdominal obesity prevalence by LASO (%) ○ | | |  |
| Characteristics | Men | P-value* | Women | P-value* | Men | P-value* | Women | P-value* |
| **Age-standardized sample†** | 22.2 (21.4-22.9) |  | 63.4 (62.6-64.1) | <0.001** | 37.3 (36.4-38.2) |  | 43.1 (42.3-43.8) | <0.001** |
| **Age groups, years** |  |  |  |  |  |  |  |  |
| 18-29 | 9.2 (8.2-10.3) | <0.001 | 41.5 (40.0-42.9) | <0.001 | 17.2 (15.8-18.7) | <0.001 | 23 (21.8-24.4) | <0.001 |
| 30-59 | 26.1 (25.0-27.3) |  | 72.3 (71.2-73.3) |  | 44.3 (43.1-45.6) |  | 50.7 (49.6-51.8) |  |
| 60 o more | 30.8 (28.6-33.1) |  | 71.8 (69.9-73.6) |  | 48 (45.7-50.4) |  | 52.1 (49.9-54.2) |  |
| **Marital status** |  |  |  |  |  |  |  |  |
| Never married | 10.5 (9.3-11.9) | <0.001 | 40.5 (38.2-42.9) | <0.001 | 19.1 (17.4-21.0) | <0.001 | 25 (22.9-27.3) | <0.001 |
| Married/Cohabiting | 25.1 (24.2-26.1) |  | 68.1 (67.2-69.0) |  | 42 (40.8-43.1) |  | 46.4 (45.4-47.5) |  |
| Separated/Divorced/Widowed | 25.2 (22.5-28.1) |  | 67.6 (65.9-69.3) |  | 42.1 (39.0-45.2) |  | 47.6 (45.8-49.4) |  |
| **Education level** |  |  |  |  |  |  |  |  |
| No formal school | 11.5 (7.6-16.9) | <0.001 | 56 (53.0-58.9) | <0.001 | 21.7 (16.4-28.1) | <0.001 | 37.7 (34.8-40.7) | <0.001 |
| Primary | 16.8 (15.3-18.3) |  | 70.8 (69.5-72.2) |  | 30.2 (28.4-32.2) |  | 51.1 (49.5-52.7) |  |
| Secondary | 21.2 (20.0-22.4) |  | 65.6 (64.3-66.8) |  | 35.1 (33.8-36.5) |  | 45.5 (44.1-46.8) |  |
| Higher | 26.6 (25.2-28.1) |  | 59 (57.5-60.5) |  | 44.3 (42.6-45.9) |  | 37.4 (35.9-39.0) |  |
| **Wealth Index** |  |  |  |  |  |  |  |  |
| Poorest | 6.5 (5.9-7.3) | <0.001 | 49.9 (48.5-51.2) | <0.001 | 14.9 (13.9-15.9) | <0.001 | 31.5 (30.3-32.8) | <0.001 |
| Poorer | 17.2 (15.8-18.6) |  | 65.5 (63.9-67.0) |  | 30.6 (28.9-32.3) |  | 44.8 (43.2-46.5) |  |
| Middle | 23.5 (21.7-25.3) |  | 68.1 (66.4-69.7) |  | 38.7 (36.7-40.7) |  | 49.3 (47.5-51.0) |  |
| Richer | 28.8 (26.8-30.9) |  | 68.1 (66.1-70.0) |  | 46.8 (44.5-49.0) |  | 46.8 (44.8-48.9) |  |
| Richest | 34.8 (32.4-37.3) |  | 66.9 (64.6-69.1) |  | 55.7 (53.1-58.3) |  | 44.1 (41.8-46.5) |  |
| **Natural regions** |  |  |  |  |  |  |  |  |
| Jungle | 15.2 (14.1-16.4) | <0.001 | 58 (56.6-59.4) | <0.001 | 28.4 (26.9-29.9) | <0.001 | 37.1 (35.8-38.4) | <0.001 |
| Mountain Range | 12.9 (11.9-13.9) |  | 56.9 (55.6-58.1) |  | 25 (23.7-26.3) |  | 37.2 (36.0-38.4) |  |
| Rest of Coast | 25 (23.7-26.3) |  | 69.2 (68.0-70.5) |  | 42.8 (41.4-44.3) |  | 49.2 (47.9-50.5) |  |
| Lima Metropolitan | 28.8 (27.0-30.6) |  | 67 (65.2-68.8) |  | 44.8 (42.8-46.9) |  | 46.1 (44.2-47.9) |  |
| **Area** |  |  |  |  |  |  |  |  |
| Rural | 8.3 (7.6-9.1) | <0.001 | 52.7 (51.3-54.1) | <0.001 | 18.3 (17.2-19.4) | <0.001 | 33.5 (32.2-34.8) | <0.001 |
| Urban | 25.7 (24.7-26.7) |  | 66.6 (65.6-67.6) |  | 42.1 (41.0-43.3) |  | 45.9 (45.0-46.9) |  |
| **Altitude** |  |  |  |  |  |  |  |  |
| 0-499 | 26.1 (25.0-27.2) | <0.001 | 67 (65.8-68.1) | <0.001 | 42.6 (41.4-44.0) | <0.001 | 46.5 (45.3-47.6) | <0.001 |
| 500-1499 | 19.6 (17.5-22.0) |  | 63.3 (61.0-65.5) |  | 33.3 (30.6-36.1) |  | 41 (38.7-43.4) |  |
| 1500-2999 | 16.8 (15.3-18.4) |  | 61 (59.2-62.8) |  | 29.6 (27.7-31.6) |  | 40.3 (38.4-42.2) |  |
| 3000 or more | 10.3 (9.2-11.5) |  | 53.7 (52.1-55.3) |  | 21.8 (20.2-23.5) |  | 35 (33.6-36.4) |  |
| **Chronic disease** |  |  |  |  |  |  |  |  |
| No | 17.4 (16.6-18.3) | <0.001 | 59.5 (58.6-60.5) | <0.001 | 31.4 (30.4-32.5) | <0.001 | 38.7 (37.8-39.6) | <0.001 |
| Yes | 37.1 (35.2-39.1) |  | 80.8 (79.3-82.2) |  | 55.9 (53.9-57.8) |  | 62.1 (60.2-63.9) |  |
| **Smoker††** |  |  |  |  |  |  |  |  |

| No | 21.8 (20.9-22.6) | 0.030 | 63.9 (63.0-64.7) | 0.256 | 37.3 (36.2-38.3) | 0.472 | 43.3 (42.4-44.1) | 0.003 |
| --- | --- | --- | --- | --- | --- | --- | --- | --- |
| Yes | 24.2 (22.3-26.3) |  | 66.2 (62.2-69.9) |  | 38.2 (36.0-40.4) |  | 49.8 (45.6-54.0) |  |

Weight specifications included the weighting factor and the ENDES sample specifications.

*P-value for chi2 test

**ANOVA test between men and women

†By WHO Population

††Smoke during the previous 30 days ATP-III: Men >102 cm; Women >88 cm LASO: Men ≥ 97 cm; Women ≥ 94 cm

- Prevalence AO ATP III 43.6%
- Prevalence AO LASO 40.6%
