# Supplementary material for: Socioeconomic inequalities in abdominal obesity among Peruvian adults
Source: PLoS One. 2021 Jul 21;16(7):e0254365. doi: 10.1371/journal.pone.0254365 (PMC8294571; doi:10.1371/journal.pone.0254365)
Supplement: S2 Table — (DOCX) [file pone.0254365.s002.docx]

| **S2 Table. Prevalence of abdominal obesity in men (n = 26,789) and women (n = 35,349) according to the administrative region of residence (ENDES 2018-2019) and based on IDF, the guidelines of the Third Adult Treatment Panel (ATP III) and the Latin American Consortium of Studies in Obesity (LASO).** | | | | | | | | | |
| --- | --- | --- | --- | --- | --- | --- | --- | --- | --- |
|  |  |  |  |  |  |  |  |  |  |
|  | Abdominal obesity prevalence by IDF (%, 95% CI) | | | Abdominal obesity prevalence by ATP-III (%, 95% CI) | | | Abdominal obesity prevalence by LASO (%, 95% CI) | | |
| Region | Overall | Men | Women | Overall | Men | Women | Overall | Men | Women |
| Amazonas | 63.7 (61.3-66.0) | 42.2 (38.6-45.9) | 85.6 (83.5-87.5) | 35.1 (32.9-37.2) | 12.2 (10.2-14.5) | 58.4 (55.5-61.2) | 29.3 (27.2-31.5) | 21.9 (19.1-25.0) | 36.8 (34.0-39.7) |
| Ancash | 75.3 (72.8-77.5) | 62.4 (58.3-66.2) | 87.2 (85.1-89.0) | 44.0 (41.5-46.6) | 18.8 (16.0-22.1) | 67.3 (64.3-70.2) | 40.5 (37.8-43.2) | 34.0 (30.6-37.6) | 46.5 (42.9-50.1) |
| Apurimac | 59.2 (56.7-61.6) | 39.7 (36.2-43.3) | 76.8 (73.7-79.6) | 30.7 (28.2-33.3) | 8.1 (6.2-10.5) | 51.1 (47.2-54.9) | 25.3 (23.1-27.7) | 16.5 (14.0-19.3) | 33.4 (30.2-36.7) |
| Arequipa | 77.5 (75.4-79.4) | 65.9 (62.4-69.2) | 88.5 (86.6-90.2) | 48.4 (46.0-50.8) | 26.2 (23.4-29.3) | 69.6 (66.7-72.4) | 45.9 (43.5-48.3) | 44.5 (41.3-47.8) | 47.2 (43.9-50.5) |
| Ayacucho | 64.2 (61.9-66.4) | 44.4 (40.9-48.0) | 81.3 (78.8-83.5) | 34.9 (32.8-37.1) | 10.1 (8.2-12.3) | 56.4 (53.2-59.6) | 29.7 (27.6-31.9) | 22.1 (19.4-25.2) | 36.3 (33.6-39.1) |
| Cajamarca | 59.8 (57.3-62.2) | 39.5 (36.0-43.2) | 78.3 (75.8-80.7) | 30.0 (27.8-32.3) | 7.8 (6.2-9.9) | 50.3 (47.0-53.6) | 23.7 (21.6-25.9) | 17.4 (14.8-20.2) | 29.5 (26.6-32.7) |
| Callao | 78.9 (77.0-80.7) | 68.4 (65.4-71.3) | 89.3 (87.2-91.1) | 50.1 (47.7-52.4) | 28.9 (25.8-32.3) | 70.9 (68.0-73.6) | 46.9 (44.7-49.1) | 43.1 (39.7-46.6) | 50.6 (47.4-53.9) |
| Cusco | 65.8 (63.0-68.5) | 48.6 (44.3-52.8) | 81.8 (79.0-84.4) | 36.3 (33.7-38.9) | 12.2 (9.8-15.1) | 58.6 (55.0-62.1) | 34.1 (31.5-36.8) | 27.4 (23.5-31.6) | 40.4 (37.3-43.6) |
| Huancavelica | 53.4 (50.8-55.9) | 34.2 (30.5-38.1) | 70.2 (67.1-73.2) | 25.2 (23.1-27.4) | 6.9 (5.3-9.0) | 41.3 (38.1-44.6) | 20.6 (18.4-23.0) | 16.6 (13.6-20.1) | 24.2 (21.5-27.1) |
| Huanuco | 60.7 (58.2-63.2) | 46.1 (42.5-49.8) | 74.3 (71.7-76.7) | 32.6 (30.4-34.9) | 13.1 (10.7-16.1) | 50.7 (47.6-53.7) | 29.5 (27.2-31.9) | 25.2 (22.2-28.3) | 33.4 (30.3-36.7) |
| Ica | 76.8 (74.6-78.8) | 66.1 (62.6-69.4) | 87.0 (85.0-88.8) | 48.1 (45.5-50.6) | 27.7 (24.6-31.1) | 67.6 (64.5-70.6) | 47.0 (44.4-49.5) | 45.3 (41.7-49.0) | 48.5 (45.4-51.6) |
| Junin | 65.7 (63.1-68.2) | 46.3 (42.4-50.1) | 83.5 (80.6-86.1) | 37.7 (35.2-40.3) | 12.9 (10.5-15.8) | 60.3 (56.6-63.9) | 30.7 (28.4-33.2) | 22.7 (19.5-26.4) | 38.0 (34.9-41.4) |
| La Libertad | 75.1 (72.8-77.2) | 61.1 (57.5-64.5) | 88.1 (85.8-90.1) | 44.9 (42.4-47.4) | 20.1 (17.2-23.3) | 67.9 (64.8-70.9) | 43.5 (41.1-46.0) | 38.0 (34.5-41.7) | 48.7 (45.5-51.8) |
| Lambayeque | 77.3 (75.3-79.2) | 68.0 (64.8-71.0) | 85.8 (83.6-87.8) | 45.6 (43.3-48.0) | 25.3 (22.5-28.5) | 64.1 (60.9-67.1) | 41.6 (39.4-43.9) | 41.9 (38.7-45.0) | 41.5 (38.4-44.5) |
| Lima | 78.7 (77.6-79.9) | 69.7 (67.8-71.5) | 87.5 (86.2-88.7) | 48.0 (46.6-49.4) | 28.3 (26.5-30.1) | 67.1 (65.3-68.9) | 45.3 (43.8-46.8) | 44.5 (42.4-46.6) | 46.1 (44.2-48.0) |
| Loreto | 63.9 (61.6-66.1) | 49.2 (45.6-52.7) | 79.4 (76.9-81.7) | 33.9 (31.9-35.9) | 14.9 (12.8-17.3) | 53.8 (50.9-56.7) | 31.0 (29.0-33.0) | 27.6 (24.6-30.8) | 34.6 (31.9-37.4) |
| Madre de Dios | 75.2 (72.8-77.4) | 62.4 (58.5-66.2) | 89.1 (87.1-90.8) | 45.8 (43.3-48.2) | 23.3 (20.5-26.5) | 70.2 (67.2-73.0) | 45.4 (43.0-47.8) | 39.8 (36.2-43.4) | 51.5 (48.2-54.8) |
| Moquegua | 82.9 (81.1-84.6) | 74.9 (71.9-77.7) | 90.9 (89.0-92.5) | 54.0 (51.5-56.4) | 32.7 (29.4-36.1) | 75.0 (72.3-77.6) | 52.8 (50.4-55.1) | 49.1 (45.7-52.6) | 56.3 (53.3-59.3) |
| Pasco | 64.5 (61.8-67.1) | 45.4 (41.7-49.2) | 82.9 (80.3-85.3) | 38.9 (36.2-41.6) | 11.0 (8.5-14.2) | 65.7 (62.6-68.6) | 33.9 (31.3-36.5) | 22.3 (19.1-25.9) | 45.0 (41.6-48.5) |
| Piura | 75.3 (73.4-77.2) | 61.8 (58.5-64.9) | 88.6 (86.4-90.4) | 44.2 (41.9-46.5) | 21.1 (18.4-24.1) | 66.8 (63.8-69.7) | 42.2 (40.0-44.4) | 37.3 (34.1-40.7) | 46.9 (43.9-49.9) |
| Puno | 65.3 (62.6-67.9) | 52.9 (48.5-57.4) | 76.3 (73.2-79.1) | 35.6 (33.0-38.4) | 15.3 (12.4-18.6) | 53.7 (50.3-57.1) | 33.7 (31.2-36.4) | 29.4 (25.6-33.6) | 37.6 (34.6-40.7) |
| San Martin | 67.4 (65.1-69.7) | 51.4 (48.0-54.7) | 83.8 (81.5-86.0) | 36.4 (34.4-38.6) | 14.8 (12.6-17.4) | 58.5 (55.5-61.5) | 32.4 (30.3-34.6) | 27.8 (24.9-31.0) | 37.1 (34.2-40.1) |
| Tacna | 84.0 (82.2-85.6) | 78.6 (75.8-81.3) | 89.2 (87.1-91.0) | 52.0 (49.6-54.3) | 32.3 (29.1-35.6) | 71.2 (68.5-73.8) | 53.2 (51.0-55.4) | 52.6 (49.5-55.7) | 53.8 (50.7-56.9) |
| Tumbes | 78.6 (76.7-80.3) | 67.9 (65.0-70.7) | 89.2 (87.1-91.0) | 48.9 (46.4-51.4) | 26.1 (23.0-29.5) | 71.7 (68.8-74.4) | 47.4 (45.0-49.8) | 43.0 (39.7-46.3) | 51.8 (48.6-55.0) |
| Ucayali | 73.6 (71.3-75.8) | 59.7 (55.8-63.5) | 87.4 (85.1-89.4) | 41.6 (39.3-44.0) | 20.1 (17.2-23.3) | 62.9 (60.0-65.7) | 38.7 (36.4-41.0) | 35.7 (32.1-39.5) | 41.7 (39.2-44.2) |

Weight specifications included the weighting factor and the ENDES sample specifications.

ATP-III: Men >102 cm; Women >88 cm

LASO: Men ≥ 97 cm; Women ≥ 94 cm
