# Supplementary material for: Socioeconomic inequalities in abdominal obesity among Peruvian adults
Source: PLoS One. 2021 Jul 21;16(7):e0254365. doi: 10.1371/journal.pone.0254365 (PMC8294571; doi:10.1371/journal.pone.0254365)
Supplement: Attachment — Submitted filename: PONE-D-20-35836.docx [file pone.0254365.s003.docx]

PONE-D-20-35836

**Socioeconomic inequalities in abdominal obesity among Peruvian adults**

Marioli Y. Farro-Maldonado, Glenda Gutiérrez-Pérez, Akram Hernández-Vásquez, Antonio Barrenechea-Pulache, Marilina Santero, Carlos Rojas-Roque, Diego Azañedo

**---------------------------------------------------------------------------------------------------------------------------------------------------**

This article aimed to analyse socioeconomic inequalities in abdominal obesity among Peruvian adults. Overall, this is an interesting topic worthy of investigation, which include large datasets. Considerable work has obviously gone into its preparation. Nevertheless, I have some comments and questions that I hope will be of assistance to the authors and think the paper could be improved with attention to some of these matters.

**Overall comments.**

1. For every study cited in this manuscript, I suggest adding detailed information on where the study was conducted and/or the study population (i.e., age, sex, etc.).
2. Many sentences (especially in the discussion section) that based on the reference was not explained properly. Please add the detail on the reference studies in the sentence to help readers understand.
3. I noticed some long sentences (e.g., Line 270-274) which is difficult to follow. Please re-phrase the long sentences and make it short and clear.

**Detailed comments.**

1. The sentence in the abstracts section should be revised to make it short but clear, especially the methods part.
2. Line 26, 38. AO and ECI was used first here, but no information what AO and ECI abbreviation stands for in the abstracts section.
3. The ‘Methods’ part in abstracts need detailed information on where the study conducted, who is the study subjects and their age range.
4. Line 30-31: “We analysed a representative sample of adults residing in urban/rural households (31 553 and 30 585 from the 2018 and 2019 dataset respectively).”
   a. Adults age? Men and women?
   b. Urban/rural household in Peru? Is it urban AND rural? Or is it urban OR rural?
   c. 31 553 and 30 585 is the total number of individuals or household? Perhaps you should add a comma in all the number to make it easier to read (31,553 and 30,585)
5. Line 43. The conclusion should be clearer. *E.g., Abdominal obesity in Peru was more prevalent among women. Socioeconomic inequality in abdominal obesity exists among Peruvian favouring the advantaged group. The inequality gap is less prominent among women, showing obesity being more common among the poor. Etc.*
6. Line 59: Which developing countries does the author refer to? Need a Reference to support this sentence.
7. I suggest adding a paragraph on information about the study settings (in the introduction or methods section), i.e., Total population, number of cities in Peru, the economy classification by The World Bank, the population health, etc. This brief background information of Peru will increase the reader’s understanding of the study subject and the contexts.
8. Line 104. ‘36 760’ is the number of individuals or household?
9. Line 105-106. Would the authors describe what is the inclusion and exclusion criteria? How many data extracted in the beginning? How many individuals are excluded?
10. Line 103-107. The explanation of the sampling procedure was very short. Please add a simple flowchart on how the study population generated from ENDES datasets.
11. Line 140-141. “To measure the socioeconomic inequality in the distribution of AO across the population grouped in wealth quintiles,”

Please rephrase the sentence here to make it easier to understand.

12. Table 1, 2 and 3. Typo in Age groups: ‘60 o more’.

I think it is better to maintain the same number of digits across all tables, whether it is 2 digits or 3 digits.

13. Table 1. No information on smoking in table 1, however, it is mentioned in the footnote (Line 193).
14. Line 194. I suggest dividing the section ‘Mean waist circumference and prevalence of AO’ into two paragraphs. The first paragraph talked about mean waist circumference and the second paragraph about AO prevalence.
15. Line 195-199. “In **both** groups, the mean waist circumference was higher in wealthy, older individuals, those who were separated, divorced or widowed, had a higher education, those living in lower altitudes, and subjects who self-reported chronic disease (p < 0.001).”

Please re-check this sentence to match the actual value in Table 1. I can see that some information in this sentence is incorrect.

16. Line 199-201. “The prevalence of AO among women had **small variations** across all the independent variables, and prevalence was **consistently** higher than men (85.1% vs. 61.1%, p < 0.001).”

Perhaps the author should re-phrase this sentence so it will be easier to understand by the reader.

17. Line 203-204. “By educational level, the prevalence of AO in men was positively linked with the level of education,”

What does the author meant by ‘positively linked’ in this sentence?

18. Line 240. “According to the IDF, at a national level, the prevalence of AO was 73.8%. When using the ATP III and LASO cut-off points, the percentage of AO decreased to 43.6% and 40.6% respectively (S1 Table).”

This sentence needs additional information e.g.: “According to the IDF *cut-off points*, the *overall* prevalence of AO *in this study* was 73.8%. When using the ATP III and LASO cut-off points, the percentage of AO decreased to 43.6% and 40.6% respectively (S1 Table)”

19. Line 250. “Having a greater wealth index, a higher education and living in an urban setting were the major independent determinants of inequality and **were positively associated with its prevalence**.”

What does the author meant by ‘were positively associated with its prevalence’? Was it meant these variables (greater wealth index, a higher education and living in an urban setting) positively associated with abdominal obesity prevalence? I do not think this manuscript studied the association between the variables and abdominal obesity prevalence.

20. Line 260. Please elaborate what kind of ‘unhealthy routine behaviours’ does the author meant here.
21. Line 261. Please also elaborate on what is the ‘ultra-processed food’.
22. Line 268, 270, 273, 281. Please mention in which country is the cited studies were conducted? This will help the reader understand the context.
23. Line 280. “The population in urban areas is 1.9 times more likely to have lower physical activity when compared to that living in rural areas [42].” If this is not the findings of this study, please add detailed information on where is the reference study was conducted etc.
24. Line 281. “It has been reported that occupational physical activity in metropolitan areas has been reduced by about 120 kcal/day [45].” Where it is reported? Who reported it?
25. Line 287-289. “Roads in rural areas are usually very rough with insufficient access ways, thus residents of this area are conditioned to do more physical activity as part of their daily commute [47]”

This sentence cites WHO report on ageing and health, is that correct?

26. Line 296-298. “This was concentrated in the 30-59 age group, a fact that could be explained both by the hormonal component typical of menopause and postmenopause [50],”

What does ‘This’ refer to? I think it is better to rephrase to “*Among women, abdominal obesity was….”.*

I also do not agree to connect this age group to menopause or post menopause. How does the author explain those women in the age group 60 or more having lower prevalence of AO? On the opposites, in the next sentences, the author mentioned about the fertility rate in the same age group.

27. Line 200-300. “We must acknowledge the large proportion of **young women** with AO compared to their male counterparts”.

Do ‘young women’ here refer to women in the age group 18-29 years old?

28. Line 301. “In Peru, women between 20-34 years of age reach the highest levels of fertility, **exceeding 60%** in both rural and urban areas [51],”

What is exceeding 60%? What is this number refer to?

29. Line 302-308. Table 1 showed that the majority of women in this study were having secondary (37%) and higher (33.5%) education level. Which means the participation of Peruvian women in the labour market may be higher. It somehow contradicts with the two sentences here. How do you explain this?
30. Line 308-309. “Finally, due to social beliefs in **some contexts**, obesity could be seen as a symbol of good economic status”, Which contexts? Does this social belief also exist in Peru?
31. Line 308-311. “Finally, due to social beliefs in some contexts, obesity could be seen as a symbol of good economic status [19], becoming widely accepted among women because of its association with pregnancy and child-bearing [55].”

This sentence should be divided into two sentences, as it talked about obesity from a different point i.e., obesity as a symbol of good economic status and obesity association with pregnancy and childbearing.

32. Line 325. “We found that the socioeconomic characteristics of the population impact the distribution of AO in Peru”.

The author can delete ‘We found that’ in this conclusion section.

The socioeconomic inequality in AO exists, but it is less prominent among women.

33. Reference.

Perhaps the author should update some outdated references to more recent publications.

Several references were not in English, and no translation made available in the Reference section.

Some references were not complete and not cited according to Vancouver style.

**---------------------------------------------------------------------------------------------------------------------------------------------------**
